# Violence in the Education: The Post-Revolutionary Situation in Hungary (1956-1957)

**DOI:** 10.12688/f1000research.140928.1

**Published:** 2023-09-28

**Authors:** Lajos Somogyvári

**Affiliations:** 1 Department of Education Sciences, Faculty of Humanities,, University of Pannonia, Veszprém, 8200, Hungary

**Keywords:** Hungary, history of violence, history of education, 1956 Revolution, armed forces, narrative analysis

## Abstract

**Background:** The author attempts to introduce an unusual approach towards schools, universities, and dormitories, including their users (professors, teachers, parents, and students): How can the educational issues be seen through the lenses of special police forces in a specific historical moment? After the 1956 Hungarian revolution, a brutal pacification process took place all over the country, supported by the Soviet troops and special armed forces, police battalions of the restarting communist power.

**Methods:** In this historical study, I used mainly military archive documents to show the perspective of the communist restauration and confront their viewpoints with party reports and some oral histories by eyewitnesses, who suffered several injuries during the repression. The paper is based on narrative analysis, as the official explanation presented various stories to justify their actions, while the reality in the background might be very different from this.

**Results:** Between November 1956 and May 1957, these soldiers or officers became a familiar image in educational institutions. They blamed teachers and professors for misleading their students and thus creating a narrative of the counter-revolution of October 1956, while they identified themselves as parents or teachers (instead of the real ones, who lost their rights to do this, due to the participation in the revolution). Conversely, physical and verbal aggression was a widespread routine of the army officers.

**Conclusions:** In extraordinary situations, during historical crises violence became suddenly real, allowed, and/or supported by many political actors to achieve their goals. After the consolidation of power, these special army and police forces were released and their activities were stopped by the authorities of the Ministry of National Defence because their presence was realized as an uncomfortable situation for the politicians. Such studies may give lessons us to learn, about how these scenes escalate into a point of no return.

## Introduction

### Process and historiography of the research

My research questions concentrate on the everyday life experiences of the post-revolutionary situation, interpretations, self-images, and justifications of different affairs in education; the basic corpus will be provided by the narratives of the armed forces ( *karhatalom, karhatalmisták* in Hungarian), supplemented and confronted oral histories of eyewitnesses, and minutes of party meetings from distinct levels. The following tabooed and traumatized stories have been hidden and latent since then; verbal and physical violence, aggression, and various forms of intervention have a multifaceted nature, showing the whole spectrum of student/teacher reactions from resistance to collaboration.

This topic is very sensitive and suppressed; it is hard to speak about it even now. There are some other written testimonies, but most of the sources came from the contemporary authentic party and military documents, which may have influenced scholars and theories by their particular language. The communist utopia started with the establishment of an ideological worldview, which transformed the categories and perception of social reality by altering the meaning of everyday words (we will see how the revolutionary days turned into a counter-revolutionary concept within just a few weeks and determined the interpretation of what happened in October-November 1956 for decades). ^1^

Even in the 2010s and 2020s, this language, when explaining the communist period, using their key ideas, without reflection, affected Hungarian historiography: it is a very good example of how the last stage of socialization has been depicted in agriculture. Many historians accept the developed discourses of the late 1950s and early 1960s (their fundamental sources), defining this radical change as a cooperative movement, meanwhile, other academics operate with the term collectivization: the former covers the intrinsic pressure and suggests a voluntary-like process, while the latter one underlines and reflects the original Stalinist initiative. ^2^ This short detour just wants to shed more light on the language of communism and its usage, which is crucial in my interpretation.

The characteristics of the records (and archives) from the 1950s and’60s demonstrate another important aspect when seeing the limitations of the usual ‘archive-based research’ in the history of education: silences and scattered references influence and form our historical reality, with the need to offer a reasonable explanation for the past. ^3^ The narratives made by the military forces and party representatives conceal some elements and emphasize others: in this case, the research was more like a puzzle solving from dispersed hints and vanished cues, because my main sources tried to erase what really happened in Hungarian education in these months, condemned forgetting the shocking details. After the end of socialism, 1956 became a central point of Hungarian self-identity and one the most important parts of our 20 ^th^-century history. Today, literature on the revolutionary events and the consequences is available in English as well: most of them introduce the geopolitical situation of the Cold War, displaying Hungary as a puffer zone between the two world orders. ^4^ Beside the politically focused works, there have been some studies closer to my approach; oral stories from the children of the executed or imprisoned victims of the retaliation, ^5^ or the voices of refugee students in a Reception Centre. ^6^ If we additionally consider Hungarian historiography, the appearance of military forces and their roles in the 1956 Hungarian education is a remarkably absent topic. There has been only one exception, which has drawn my attention to the special armed troops visiting schools armed groups and their activities. ^7^ This paper, which started with a lost and found dossier by me in the National Archive in 2016, meets my research interests.

## Methods

### Ethical considerations

In this case, we face a sensitive and complex socio-historical issue – by describing it, I followed two main principles. First, it is crucial to commemorate these tabooed events and prevent to forget them. On the other hand, I do not featured concrete names in the analysis (except of the widely known politicians, as Kádár) to give respect for the people who were affected and follow the actual Hungarian legislation on the right to informational self-determination and on the freedom of information (Act CXII of 2011, see: https://net.jogtar.hu/jogszabaly?docid=a1100112.tv). This study received ethical approval from the Ethics Committee of the University of Pannonia, Hungary. I do not utilize personal stories or interviews – that is why I focused only on the archives and the published oral histories, *ad perpetuam rei memoriam.*

### Data sources

Unforeseen documents popped up when I made investigations about denominational instructions in the academic year of 1956-1957: a file, entitled K. A. (likely the capital letters of the then head of Educational Ministry, Albert Kónya), which contained records about teacher beatings, rude interrogations, and threatening incidents. ^8^ This was a lucky coincidence, as this folder has been totally unknown in the Hungarian historiography, which I found accidentally in fragmentary documentation – many files were destroyed during and after the 1956 revolution. In the next step, I gathered the official Party archives on a national level, searching traces of violence in schools after October 1956: more and more details emerged, related to the activities of special armed forces, included in my first paper on the subject. ^9^ Following this logic, I analyzed the historical logs of these regiments and battalions in the Hungarian Military Archive, ^10^ and contrasted the official discourses with two retrospective personal testimonies from public oral histories. ^11^ There are two main, complete corpora as the basis of my study, the first one is the paper collection of Albert Kónya, who was a deputy minister and provisional head of the Educational Ministry at that time. His documents are stored in the Hungarian National Archive, Section of Culture, Educational Ministry (MNL OL XIX-I-2-j), from our point of view the fourth box (4. d.) is the most important. To start research here, you have to apply, signing for your purpose (private or scientific), topic, and time period. It is open and free for every Hungarian and non-Hungarian citizen, the application form and all possible regulations and restrictions are readable here: https://mnl.gov.hu/uj_kutatoknak. ^12^ Accessing to the second type of sources, a similar process needed (application, including purpose, topic and time) at the Military Archive ( https://militaria.hu/adatb/leveltariuj/). There is a thematical compilation here, called the Hungarian People’s Army Special Collection (MN KGy, Magyar Néphadsereg Különgyűjteménye), including papers about the 1956 revolution and restoration of the communist power afterward (F. 1956-os gyűjtemény, 1956 collection). ^13^ All other materials used in the paper do not constitute a group like the previous ones, they are separate documents, scattered references, that add official and non-official interpretations of the verbal and physical aggression between 1956 Autumn and the Spring of 1957: meeting of the Hungarian Socialist Workers’ Party (HSWP) and personal memories about the events.

**Figure 1.**
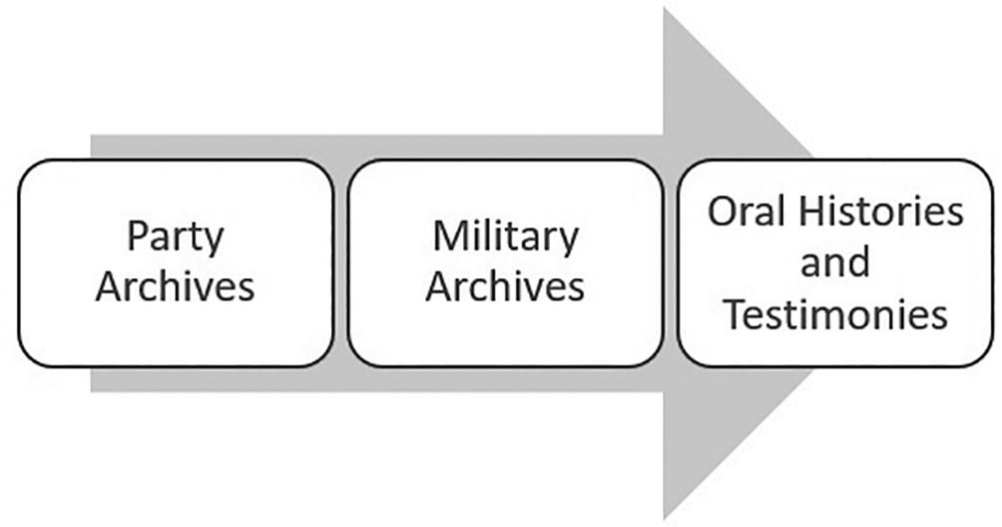
The three step-research process related to the sources.

The co-occurrence of the actors (armed forces and people who were involved in the education) and their activities with a certain degree of aggression and reaction (violence, resistance, collaboration, etc.) are the key features of my paper, which provided the sources to scrutinize my topic. Following the three-step research (see Figure 1), I will answer the following research questions by using this collection of data:

(1) How can the connections between armed forces and educational actors (teachers, principals, lecturers, students and parents) be characterized?
(2) What kind of police and special force interventions took place in the educational institutions?
(3) What image of educators, educational roles and the responsibility of the youngsters and adults do the declarative narratives of the power depict?
(4) What face of the enemy emerged from the sources?

All of these aspects are inseparably intertwined in the search of guiltiness for the heart-breaking and shameful events in October (a euphemistic definition of the 1956 first revolution, then counter-revolution) and finding accountable persons to judge over. ^14^ My analysis is based on the military archives, offering a special point of view, adding other perspectives and alternatives to consider as well (to provide balance).

**From sources to methodology**

The files of the deputy minister, Military Archives, and the Party meetings show us the basics of the official understanding of the extreme clashes in education. They had different accents and intentions during these months: the bodies of the state party tried to take pacification under control (more or less successfully) on every level, while the special forces often crossed the legal borders as emissaries of people’s power, agents of real communism. The different goals caused friction and conflicts between party secretariats, police captains, and Soviet and Hungarian colonels; the relevant actors during the stabilization period. Committees of the Hungarian Socialist Workers’ Party (HSWP) had to fight on two parallel fronts, namely, against the extreme, radical leftists, who wanted to get revenge for the revolution (from their point of view counter-revolution), on the other hand, against reformer communists and remaining followers of the failed revolt from the right side of the political palette. The armed/police forces were close to the former side, released by the power in a purposely uncontrolled situation to force discipline and order on society.

The oppressed and occupied country did not have a voice to express their opinion and feelings, which casts a long shadow on the everyday Hungarian perception and remembrance of 1956 and 1957. Invisible violence ^15^ was a substantial element of the existing socialism: experience of suppression and atmosphere of real hard-core communism inherited from generation to generation. In the long years after 1956, the legitimation of the new Kádár-regime was based on a historical compromise: ^16^ The ruling power wanted every citizen to forget what had happened, and in exchange, it offered a limited opportunity for material prosperity. The only official requisition was remaining as apolitical and neutral as possible, avoiding the dogmas and taboos of the political power, like the unquestionable statement about counter-revolution, and neglecting any other meaning of that October. If you have a discussion with an elderly person from your audience after a conference speech, and he or she shares his or her own memories about going to school in Hungary in late 1956 and early 1957, this was to be shared with you only, privately and nothing else. This can be a long-term consequence either of this previous cultural tradition or of frustrating and hurting emotions from those months. The partly hidden and latent nature of past events and the psychological distress connoted with them made oral histories very difficult, so in my research, I am only adding two testimonies from public collections of eyewitness accounts: Oral History Archive for teachers, and memories from a well-known journalist’s webpage, both published on the internet, accessible for everyone. ^17^ These descriptions tell the commonly known events of the revolution and vengeance from a specific perspective: history from below. ^18^

Military archives covering every territory, give a complete overview of the whole country because when the special forces were withdrawn, their commandants had to outline the activities related to law enforcement in the form of historical logs. All of these logs remained and microfilmed, ^19^ which is a very good starting point to the analysis, while other sources (party documents, meetings, reports, interviews, etc.) are very fragmented and concerned with particular issues. However, the latter data also underpins the whole picture. To set the framework of this undiscovered historical field, using the theoretical background of emotions and violence in history can be fruitful here, ^20^ in order to present meanings and contexts of abusing power. Every element of this investigation is unique: the chosen topic, the archives, and the nature of the sources, which all pose challenges in the methodology. I focused on the textual appearances of schooling, students, and teachers, including their relations with the armed forces. By doing so, the main topics of these archives could easily be detected, these are the following: interpretation of the revolution and resistance, activities to restore communist power and legitimizing the actions and brutalities during the retaliation. Reconstructing the official argumentations and stories was the last step in the research.

The study is based on narrative analysis, because one obvious characteristic of the examined army logs is narrativity: they told us stories with traditional elements, like conflict, heroes and enemies, problem-solving, happy end, and so on. The writers (military leaders) wrapped up the pacification process as a success, their ‘emplotment’ produced familiar literary genres, with enough ‘explanatory power’ ^21^ to give plausible explanations for the resistance in society – both they and their political supervisors needed this. Revolution meant collapse and chaos, a time of uncertainty to the Party apparatchiks and loyal soldiers, a kind of tragedy, followed by the triumph of the good (a romance), the victory as a restoration of the communist power.

According to Eileen H. Tamura, ^22^ to understand historical reality and the human agency within, it is essential to consider individual events, which is logical in an idiographic discipline. The narratives provided by the heads of special armed/police forces contain two main organizing principles:

(1) scapegoat-theories,
(2) and the takeover of educational roles from teachers and professors.

From their point of view, it was a rational and logical decision: many educators were involved in (counter-) revolutionary activities; they became responsible for every later opposition, and thus, lost their rights to instruct the younger generations. The battalion commanders, lieutenant colonels, and officers started acting as quasi-educators in three dimensions, laid down and transmitted in the reports to the superior bodies of the HSWP. They:

(1) are the ones to guide misled students, instead of original teachers,
(2) regulate principles, leaders and faculty staff,
(3) threaten parents to hold down their children.

There might have been many psychological factors in the background of verbal and physical aggression (individual motives, fear and hate, the feasibility of a new rebel, group pressure, anti-intellectualism, etc.), but I will not examine these, as they are not my focus, and they can be found in the sources. The next chapters will detail the developing relationships between armed forces and educational actors through diverse interventions in the everyday life of schooling.

## Results

### Arrest and interrogation

On 12 December 1956, at 4 a.m., a secondary high school teacher was arrested in Eger, a small Hungarian city, in his home. ^23^ There were two soldiers, one detective, one driver, and an authority-appointed witness; the scene must have been very similar to how Solzhenitsyn illustrated a typical arrest during Stalinism; “The sharp night-time ring or the rude knock at the door. The insolent entrance of the unwiped jackboots of the unsleeping State Security operatives. The frightened and cowed civilian witness at their backs.” ^24^ The teacher was accused of hiding weapons by an anonymous denunciation. The armed forces unit did not find anything, yet the arrest and imprisonment of the teacher was inevitable. Weeks later, his wife still had no answers because of the demand for ‘military dictatorship’ no information was distributed; but according to the appendix of the document, ^25^ he was released in early 1957. The whole faculty staff stood up for him; they wrote a letter of solidarity, stating his innocence – all of them signed the statement, which testified to extraordinary courage at that time.

Sometimes teachers were called to the municipality during worktime, as in an elementary teacher’s case, in a village near the Romanian border in February 1957. ^26^ When he entered the official room, his ID card was expropriated, then (without any question) the police officers began to beat him; he got slapped, and his hands and other body parts punched with a stick. It was accompanied by hateful comments (verbal and physical aggression usually went hand in hand): “Did you want to demolish the power of people? We will give you power! (…) So far, there has been no dictatorship, now there will be the dictatorship of the proletariat!” ^27^ After some hours standing in a dark cell, he was let go, but banned from every school in Hungary, because “a counter-revolutionary teacher doesn’t have the right to teach”. ^28^ His only crime was finalizing the minutes of the local revolutionary committee in October 1956. There are several other documents proving the ubiquity of such incidents: teachers feared removal, arrest, and beating; ^29^ the Party apparatchiks and workers condemned them as a whole. ^30^ Furthermore, principals were frequently subjected to extreme physical abuse, without any hearing. ^31^

If there was a hearing, it often had a very intimidating atmosphere. For example, the police captain in Szeged said the following sentences to university students after they had been taken into custody: “The university buildings will be shot up, from the basement to attic! (…) All professors are fascists; they only joined the Party to hide their medals as former members of the Order of Vitéz”. ^32^ A universal topos appeared here: teachers and lecturers are the successors of the Horthy era (this Order was a creation of that regime), an emblematic period of fascism before 1945, which was a misleading simplification of communism. Other officers defined the “bloody” professors as “pimps”, the armed forces would “take care of them” – these expressions were deeply emotional, and full of negative attitudes. ^33^

### Looking after guns and leaflets

House searches, framed as inspections, meant another important interventional form of the special forces as a following symptom of arrest, or as independent activities. The most well-detailed incident happened at Miskolc University on 20 February 1957: we have four sources that day, from different perspectives, ^34^ which gives us an exceptional opportunity to dig deeper. In the report of the notorious ‘Tizeshonvéd’ zászlóalj (Battalion no. 10.) ^35^ self-justification, legitimacy of action, and euphemism amalgamated: 300 officers were involved in the attack, which was caused by a scandal. According to the rapporteur, the students humiliated the flag of the international working class by hanging out red rags, and women’s underwear on it from the windows of their hostel, answering the official flag of the main building. The armed forces made a blockade around the student hostel, and started the search: they found guns, leaflets, and confidential papers of the State Security (ÁVH); while the youngsters mocked the officials (“bloody Kádár-soldiers”, whistled the Soviet hymn ironically), and behaved so “arrogantly and shamelessly”, that they were scolded like misbehaving children. ^36^ There is no explanation as to what is meant by the last comment.

The rector’s memorandum provides more information: 60 students and 10 lecturers were arrested; many were beaten during the search, including the vice-rector, a dean, and some students “were very sick” ^37^ because of the thrashing. The leader criticized both the provocation by the students (the red rags) and the violation of law by the armed forces. ^38^ The scandal reached the highest levels of the party bodies. In a meeting of the HSWP Organisational Committee, the party secretary of Borsod briefly recapped the events: the soldiers tried “to correct the thinking [of students] with some slaps (…) Professors are offended because they were beaten too …”. ^39^ An oral narration from a former student remembered this way:

“… we had to stand by the wall, with our hands behind us. And from then on, they started beating and beating us. (…) they took the nightsticks and banged. Then other officers came, asked our names and occupations of our fathers, and depending on the answer, they began to hit again. Most of us were from the working class in Miskolc (…) which aroused wild rage in the soldiers because they could have been the same (…) laborers; when they heard “engine fitter”, they said “You m … f … er”, and began to beat.” ^40^

The quotation reflects to a very important factor of the communist systems: the determining class, ^41^ the social background that determines everything from schooling to employment; and the whole identity of a citizen in Hungary. From the official point of view, every (counter-) revolutionary person had a bourgeois, aristocratic, and fascist heritage, which connects the rebels with the previous Horthy Period (1920-1944), but reality always refuted this preconception, because the majority of the freedom fighters were from the working class. This schizophrenic situation (insisting on the ideology, as opposed to people’s own experiences and made-up interpretations) caused a lot of stress and frustration among the loyal members of the new power and resulted in a spiral of violent acts.

A slogan was spreading very fast in early 1957: ‘We will start again in March’ (in Hungarian: Márciusban Újra Kezdjük, in short: M.Ú.K.). It referred to the national commemoration of the 1848 Revolution (March 15) with the oppressed revolt and suggested a new uprising. More and more affairs refer to the probability of such an event, but we do not know much about the real background of such a movement (there are studies that stated that issuing this idea was the initiative of the power, in order to strengthen its position). ^42^ For instance, in Kecskemét downtown, shop windows were inundated with handmade leaflets and posters before 15 ^th^ March, with the mysterious signatures KEFI and TBSz. The secret police made a covert action to unveil delinquents, and a fewdays later, they uncovered a ‘conspiracy’ at the Piarista Gimnázium (Piarist Secondary School), a denominational institution, which was a lucky coincidence for the authorities, as a good illustration of the entwined clerical-fascist assault against people’s democracy. ^43^ The fliers and placards were produced by 11 students. The detectives diagnosed that only one of them was of laborer origin, the others were sons of lawyers and previously leading functionaries, which confirmed the bourgeois character of their propaganda activities. They constituted the Revolutionary Youngsters of Kecskemét (Kecskeméti Forradalmi Ifjúság, KEFI), also known as Turul Brotherhood (Turul Bajtársi Szövetség, TBSz) ^44^ – the latter one invoked a radical interwar right-wing organization, but we do not have more information as to whether a real continuity existed between them.

There was a symbolic fight in these months about ruling the streets and public opinion: these propaganda messages occupied the public sphere, which originally fell within the scope of the party and its ideology after 1945, and operated in short impressive slogans. ^45^ The longstanding social resistance borrowed this instrument from communists in order to score the victory over them (at least) symbolically when the making and distribution of posters and leaflets remained the last battlefield. Teacher trainees majoring in art created posters in Szeged, but they were caught in the act while gluing; after that, 30 police officers in civil clothes, the armed forces, and the Party Committee made joint raids on Fridays and Saturdays for three weeks. ^46^ In Borsod County, anti-Soviet poems and handbills spread in all schools: “We do not learn for Kádár”, “Russians go away”, “We will not learn the Russian language”, and “Our leader is Mindszenty”. ^47^

### Administrative actions and information work

The title includes two key phrases from the vocabulary of armed forces: administrative actions meant removals from schools, under the term ‘information work’ including political debates, meetings, and briefings arranged to give parents, students, teachers, and principals a kind of ideological indoctrination. Schools were evaluated as centers of the counter-revolution in the papers of the 1 ^st^ Regiment of Revolutionary Armed Forces, ^48^ and they set the goal to purify the institutions of the negative spirit: for example, a girls’ schoolteacher was arrested, because she had educated children in an anti-democratic spirit. ^49^ A compelling dichotomy ^50^ defined the school perception of army/police officers: some teachers and students personified the face of the enemy as agitators of the counter-revolution, warmongers who risked peace. On the other hand, there were loyal socialist citizens, supporters of the workers’ state, and many hesitating people. There was no mercy on the first group; meanwhile the latter ones had to be strengthened further: thus school-visiting groups were formed, who involved parents and teachers with communist confidence, principals, and school departments in the work.

These groups used intimidation and persuasion as their tools: teaching staff was made responsible for every conflict in schools; so out of fear or conviction, they reported everything to the battalions. The armed forces participated in the discussions between form teachers and students, as in parent-teacher association (PTA) meetings, they warned parents to protect their children from the risks of counter-revolutionary activities (according to the accounts, ^51^ they did so in an objective, rational manner, which is doubtful). Principals did not allow these gunmen to work in every school (a clear sign of a living struggle), as the troop leader of the 7 ^th^ platoon faced this issue and answered in agreement with the Party: “We have to get into the schools and classrooms”. ^52^ The units made lesson plans to analyze topics with children, with titles like “What is the meaning of Kossuth-crest?” [an old traditional Hungarian symbol and a chosen crest of the 1956 Revolution], “The significance of the red flag and red star”, and “What are the pieces of evidence that happenings in October were counter-revolutionary?”. Soldiers and police officers taking the role of teachers by giving lectures and instructions declared that every educator is obliged to act adopting the official standpoint of the government, “even if they had political concerns”. ^53^

Helping to build pioneer patrols, establishing Communist Youth League (Kommunista Ifjúsági Szövetség, KISZ) and Youth Guard (Ifjú Gárda), restarting Party in teaching staffs, planning trips, and leading cultural centers – these are just some activities associated with the armed forces. The operative, secret police work was more hidden, the only certain point is that every battalion had to assign liaison officers to do this in Budapest (no information outside the capital city). The sources mentioned such activities two times:

“The use of children of communist parents in exploring counter-revolutionary elements proved to be a very good method. These children told us what some teachers had incited them to do, which classmates had boasted that they or their parents possessed guns illegally.” ^54^

“A young, 17-year-old boy offered us to help catch a person who was actively engaged [in the events of October], and advertised to others that he was working for the secret police …” ^55^

The functioning system followed the ancient political phrase of ‘ *Divide et impera’* (divide and rule): thus they separated different groups and confronted them with each other; the pure form of educational micropolitics ^56^ intertwined every situation and context. Another 17-year-old student was arrested in Veszprém on 29 April 1957, with the charge of conspiracy against the people’s democracy. The beatings lasted for days; his eardrum broke in, and the interrogators wanted him to speak about the ‘Young Hungarian Alliance’, a naïve local teenager movement, which was planned to start again in March (not successfully) and distribute leaflets. He refused to make a confession. Six days later, they let him go, but one week later, arrested him again. ^57^ The forms of punishment applied were diverse: slaps, beaten by a baton, and gymnastics (squats and pushups) repeatedly on a circular basis, and they also threatened him by being banned from every secondary school in Hungary (that was a real punishment at the time). The only way out was if he signed a statement about future cooperation with the police. After two days of punishment, he signed the paper, admitted the counter-revolutionary propaganda, and offered his services to state security to avoid punishment. After his release, he never went to the meetings with officers, and the connection was disrupted when he moved to Budapest. ^58^

A very different narrative speaks about officials and students’ relationship in the case of the university dormitory in Buda, District I. ^59^ The historical log of the 3 ^rd^ Regiment provides a whole story, with a plot of how misled students got directed in the right direction by the officers. The start is a confusing period when the resistance was alive in January 1957. The site was the Buda Castle District, where several underground basements provided ways to hide and store illegal materials, and to transport them from one place to another. The armed forces raided there many times, putting students under pressure by threatening them with issuing a shooting order, without relevant results, so new methods were necessary. The 1 ^st^ Battalion arranged a dance event for the university students, under strict control: another typical educational activity turned into a bizarre scene, where gunmen surrounded the dancing pairs.

On 23 February 1957, at midnight, a meeting was being held between a lieutenant colonel, a first sergeant, a first lieutenant, and the community of the dormitory. The situation described looked like a parents-children discussion, full of protection and intimacy literally in the form of a dialogue:

“Student: ‘Comrade Lieutenant Colonel! We asked you to forget the past! (…)

Lieutenant Colonel: ‘We are angry with you, like good fathers (…) Do you take any steps to get closer to us?

Student: ‘Please, trust us in the future!

Lieutenant Colonel: ‘You have to deserve it first.’” ^60^

The following topics shaped the political debate from 7 p.m. to 2 a.m.: the Miskolc University scandal, the role of the armed forces, the events in October as a revolution, the political roles of the university students, etc. The youngsters were very critical and well–informed; they had not acquired the concept of counter-revolution, but the end of the story is a happy ending from the viewpoint of the armed forces. Some university students were convinced at the meeting, and they later visited secondary high schools with the officers to convey the message: “Our friends, fellow students – we were deceived!” [by the counter-revolutionists, the enemies of the communist system, and the people’s state]. ^61^ From the viewpoint of the armed forces, after March, their victory was fulfilled – from the opposition to achieving the common goals; it was a long development history.

## Conclusions

The 6 ^th^ Battalion of the 2 ^nd^ Regiment summarized the significance of these activities: “The enemy attacked through schools”, ^62^ from the viewpoint of the churches and the counter-revolution, therefore a definite change toward the true way of building socialism was brought about. In previous years, some teachers educated children improperly, so “we had to take the issues of youngsters in our hands”. ^63^ From a retrospective point of view (June 1957), it was a triumph, but without the help of communist officers and loyal teachers, this progress could not have been imaginable. A chaotic period emerged from the sources, a whole spectrum of possible practices, and contexts changed over time, meanwhile the official discourses tried to suggest a planned evolution and a coherent argument about the reasons of the (counter-) revolution. The armed forces existed only for a limited time of the communist takeover between November 1956 and June 1957: first, the politicians needed this power. After the stabilization, they became an unnecessary ballast that had to be terminated. In the light of recent developments (e. g. Russian aggression against Ukraine, children faced acts of war, violent environments of schooling) the topic of my study is very relevant. Perhaps learning from the past, and reading how these situations developed is a big benefit of such analyses.

## Data Availability

The underlying data for this study is under restriction by the Archival resources of the Hungarian National Archive (MNL, Magyar Nemzeti Levéltár, https://mnl.gov.hu/) and the Military Archives (HL, Hadtörténelmi Levéltár, https://militaria.hu/adatb/leveltariuj/). To request access to the same data used in this study, researchers should contact to these institutions, the e-mail addresses are the following: info@mnl.gov.hu (Hungarian National Archive), and kutato@mail.militaria.hu (Military Archives). In the case of the Hungarian National Archive, a pre-registration is required with a full name and e-mail address ( https://www.eleveltar.hu/auth#step=1), after a successful registration one can submit a request in the electronic database ( https://www.eleveltar.hu/) with the precise number of the document (like XIX-I-2-j. 4. d. in this paper). Filling a Supporting Statement and a Visitor Ticket (Támogatói nyilatkozat, Látogató jegy, here: https://m.militaria.hu/adatb/leveltariuj/content/letoltheto_urlapok_kutatoszolgalathoz_es_kozhivatali_tevekenyseghez ) is needed to access to the files in the Military Archives. The Supporting Statement has to be signed and stamped by a scientific institution, indicating the subject and timeframe; the Visitor Ticket included your personal data (name, date and place of birth, mother’s name, address), and the purpose of the research (private or scientific). All of these forms are in Hungarian, but you can ask for help by writing an e-mail to the Research Office ( kutato@mail.militaria.hu).

## Footnotes

^1^
S. Kotkin, *Magnetic Mountain: Stalinism as a Civilisation* (Berkeley: University of California, 1995); J. Hellbeck, *Revolution on My Mind: Writing a Diary Under Stalin* (Cambridge–London: Harvard University Press, 2006); and I. Paperno, *Stories of the Soviet Experience: Memoirs, Diaries, Dreams* (Ithaca–London: Cornell University Press, 2009). These compelling works illustrate the perception of the communist world, the discrepancy between official and private languages and different identity-making mechanisms in many ways.

^2^
S. Horváth and J. Ö. Kovács, ed., *Állami erőszak és kollektivizálás a kommunista diktatúrában* [State Violence and Collectivisation in the Communist Dictatorship] (Budapest: MTA BTK, 2015).

^3^
G. McCulloch, *Documentary Research: In Education, History and the Social Sciences* (London-New York: Routledge, 2004), 44-45. McCulloch cited here Carolyn Steedman’s lines about archives and their emotional, physical effects on historians; here, one goal seems to be ubiquitous: to give justice to the voiceless and forgotten people who were oppressed in 1956 and 1957 in Hungary.

^4^
Cs. Békés, M. Byrne and J. M. Rainer, ed., *The 1956 Hungarian Revolution: A History in Documents* (Budapest-New York: Central European University Press, 2002); J. Granville, *The First Domino: International decision making during the Hungarian Crisis of 1956* (College Station: Texas A&M University Press, 2004); C. Gati, *Failed Illusions: Moscow, Washington, Budapest, and the 1956 Hungarian Revolt* (Washington-Stanford: Woodrow Wilson Center Press and Stanford University Press, 2006); J. M. Rainer and K. Somlai, ed., *The 1956 Hungarian Revolution and the Soviet Bloc Countries: Reactions and Repercussions* (Budapest: The Institute for the History of 1956 Hungarian Revolution and Historical Archives of the Hungarian State Security, 2007).

^5^
Zs. Kőrösi and A. Molnár, ed., *Carrying a secret in my heart … Children of the Victims of the Reprisal after the Hungarian Revolution in 1956. An oral history* (Budapest-New York: Central European University Press, 2003).

^6^
V. Sheridan, ʻSupport and surveillance: 1956 Hungarian refugee students in transit to the Joyce Kilmer Reception Centre and to higher education scholarships in the USA’, *History of Education* 45, no. 6 (2016): 775–793.

^7^
R. Maruzs, ʻKülönleges feladatok a karhatalomban [Special roles in the armed forces]’ in *A dolgozó népet szolgálták? Fegyveres testületek és pártirányításuk a szocializmusban* [Did they serve the working people? Armed forces and their control by the political party in state socialism]. Eds. I. Ötvös and P. Triber (Budapest: NEB, 2018), 67–82.

^8^
Magyar Nemzeti Levéltár Országos Levéltár (MNL OL) [Hungarian National Archive] XIX-I-2-j. 4. d.

^9^
L. Somogyvári,’”Eddig nem volt diktatúra, most lesz proletárdiktatúra!” Rendteremtés a pedagógus-társadalomban 1956 után [”So far, there has been no dictatorship, now there will be the dictatorship of proletariat!” Pacification of teachers after 1956]’ in *A tanulás új útjai* [New ways of learning]. Ed. J. Mrázik (Budapest: HERA, 2017), 620–633.

^10^
L. Somogyvári, ‘A karhatalom vidéken: iskolák, egyetemek és diákszállók az 1956–1957-es tanévben [Armed forces in the countryside: schools, universities and dormitories in the 1956–1957 semester]’ in *Jövőformáló human tudományok* [Humanities in the service of the future]. Ed. J. Tóth (Budapest, Akadémiai Kiadó, 2022), 114–121.

^11^
Tanári kézikönyv az 1956-os forradalom történetét feldolgozó SULINET digitalis órához [Teachers’ handbook to the digital SULINET lesson about the 1956 revolution]. Retrieved from: https://mek.oszk.hu/01900/01937/html/szerviz/. Borenich Péter honlapja [Webpage of Péter Borenich]. Retrieved from: https://borenichpeter.hu/

^12^
The text is in Hungarian, but the Information Office at the Archive can help everyone to fill the form.

^13^

To find these sources, there is a guidance here: https://militaria.hu/adatb/leveltariuj/f/08_viii_xiii_fondfocsoport_magyar_ nephadsereg_iratainak_segedletei

^14^
Az MSZMP Ideiglenes Központi Bizottságának határozata, 1956. december 5. [Decree of the HSWP Provisional Executive Committee on 5 December 1956] in *A Magyar Szocialista Munkáspárt határozatai és dokumentumai, 1956-1962* [Documents and Resolutions of Hungarian Socialist Workers’ Party, 1956-1962]. Eds. Á. Ságvári and H. Vass (Budapest: Kossuth, 1964), 13–17. This document defined the evaluation of the 1956 revolution as a counter-revolution until 1989: counter-revolution had to understand as an essential attack against the workers’-farmers’ state (the real revolutionary organisation) after December 1956. In short, the main reasons of the rebellion are: two opposite poles of the Party (the dogmatic, sectarian, Stalinist clique, and the revisionist, right-wing group of Imre Nagy), the Horthy-fascism, and of course, the international imperialism.

^15^
J. Baberowski, *Räume der Gewalt* (Frankfurt am Main: Fischer Verlag Gmbh, 2015), 110–132.

^16^
B. Kovrig, ʻHungary in the era of the Kádár compromise’, *International Journal* 33, no. 6 (1978): 720–739.

^17^
Tanári kézikönyv az 1956-os forradalom történetét feldolgozó SULINET digitalis órához [Teachers’ handbook to the digital SULINET lesson about the 1956 revolution]. Retrieved from: https://mek.oszk.hu/01900/01937/html/szerviz/. Borenich Péter honlapja [Webpage of Péter Borenich]. Retrieved from: https://borenichpeter.hu/

^18^
K. Myers and I. Grosvenor, *Collaborative Research: History from Below* (Bristol: University of Bristol/AHRC Connected Communities Programme, 2018). This fascinating book underscores the role of communities and the importance of participation in such researches: the everyday perception of communism very much differs from what we have known from the usual sources. For example, one of my projects aims to reveal the schooling experience of the so-called ʻclass-alienate’ children, who were not allowed to enter secondary and higher education in the 1950s because of their social class (former aristocrats, sons and daughters of the members of gendarmerie, etc.), to shed a light on the situation. We are not able to find these stories in the archives, only in oral sources.

^19^
Hadtörténelmi Levéltár (HL) [Military Archives] MN KGY, F. 10. d., 11. d. There is a selection called: Magyar Néphadsereg Különgyűjtemény (MN KGY), F. 1956-os gyűjtemény [Hungarian People’s Army Special Collection, 1956 collection]. 10. doboz (Budapest), 11. doboz (vidék) [Box 10. Budapest, Box 11. Countryside].

^20^
B. H. Rosenwein (2002). Worrying about Emotions in History. *American Historical Review* 107, no. 3 (2002): 821–845.; N. W. Sobe, ‘Researching emotion and affect in the history of education’ *History of Education* 41, no. 5 (2012): 689–695.; C. Tilly, *The Politics of Collective Violence* (Cambridge: Cambridge University Press, 2003); C. Veiga,’The body’s civilisation/decivilisation: emotional, social, and historical tensions’ *Paedagogica Historica* 54 no. 1–2 (2018): 20–31.

^21^
H. White, *Metahistory: The Historical Imagination in Nineteenth-century Europe* (Baltimore-London: The Johns Hopkins University Press, 1973). The categories (like tragedy, romance, modes of arguments) of this influential work fit very well in my sources, which may be an evidence of the literary abilities of the colonels.

^22^
E. H. Tamura, ʻNarrative History and Theory’, *History of Education Quarterly* 51, no. 2 (2011): 150–157.

^23^
ʻKónya Albert felterjesztése’ [Memorandum of Albert Kónya] XIX-I-2-n. 8. d. 123/1957, 14 February 1957, MNL OL.

^24^
A. I. Solzehnitsyn, *The Gulag Archipelago, 1918-1956. An Experiment in Literary Investigation* (New York: Harper-Perennial Classics, 2002), 4.

^25^
‘A tanári kar nyilatkozata’ [Statement of the teaching staff], In ʻKónya Albert felterjesztése’ [Memorandum of Albert Kónya] XIX-I-2-n. 8. d. 123/1957, 14 February 1957, MNL OL.

^26^
ʻJelentés az 1957. évi február 20-i Békés megyei kiszállásról’ [Report on a trip in Békés county on 20 February 1957] XIX-I-2-j. 4. d. 21 February 1957, MNL OL.

^27^
Ibid.

^28^
Ibid.

^29^
ʻJelentés a pedagógusok közt végzett politikai és szakszervezeti munkáról’ [Report on political and trade-union work amongst teachers] MSZMP KB APO [Department of Agitation and Propaganda at the Central Committee of HSWP], M-KS 288-22, 21 March 1957, MNL OL.

^30^
ʻTájékoztató a KB Agit. Prop. Osztályon tartott értekezletről’ [Information about the Session at the Central Committee Agitation and Propaganda Department] MSZMP KB APO, M-KS 288-22, 12 June 1957, MNL OL.

^31^
ʻFeljegyzés Kónya Albert művelődésügyi kormánybiztos elvtárs részére’ [Notes to Comrade Albert Kónya, commissioner for education] XIX-I-2-j. 4. d. 25 February 1957, MNL OL.

^32^
ʻMelléklet – Jegyzőkönyv Nagy Imre orvostanhallgató bejelentéséről’ [Appendix – Minutes of the notification of Imre Nagy medical student] XIX-I-2-j. 4. d. 3571/1956, MNL OL.

^33^
Ibid.

^34^
A miskolci „Tizes honvéd” karhatalmi zászlóalj története [The history of Miskolc Battalion no. 10] HL MN KGY F. 1956, 11. d. 21. ő. e.; ʻFeljegyzés Kállai elvtárs részére’ [Notes to Comrade Kállai] XIX-I-2-j. 4. d. 26 February 1957, MNL OL; MSZMP Szervező Bizottság ülése [Meeting of the HSWP Organizational Committee] KS-288-6. 30 December 1956, MNL OL; R. Ungváry, ʻA megtorlás Miskolcon’ [Retiliation in Miskolc] Oral History Archive, Interview no. 120., made in 1987-1988. Retrieved from: http://mek.oszk.hu/01900/01937/html/szerviz/oha/ungmeg.htm

^35^
A miskolci „Tizes honvéd” karhatalmi zászlóalj története [The history of Miskolc Battalion no. 10] HL MN KGY F. 1956, 11. d. 21. ő. e.

^36^
Ibid.

^37^
ʻFeljegyzés Kállai elvtárs részére’ [Notes to Comrade Kállai] XIX-I-2-j. 4. d. 26 February 1957, MNL OL.

^38^
Ibid.

^39^
MSZMP Szervező Bizottság ülése [Meeting of the HSWP Organizational Committee] KS-288-6. 30 December 1956, MNL OL. The original location is not here (see the date), this document was lost, intentionally or accidentally – every level of archival collections is incomplete in the year of 1956 and 1957 in Hungary.

^40^
R. Ungváry, ʻA megtorlás Miskolcon’ [Retiliation in Miskolc] Oral History Archive, Interview no. 120., made in 1987-1988. Retrieved from: http://mek.oszk.hu/01900/01937/html/szerviz/oha/ungmeg.htm

^41^
S. Fitzpatrick, ʻAscribing Class: The Construction of Social Identity in Soviet Russia’ *The Journal of Modern History* 65, no. 4 (1993): 745-770.

^42^
Zs. Krahulcsán,’MUK és HUKUK. A politikai rendőrség és az 1957. tavaszi „provokációk”’ [We will start again in March and if you start again, you will get again. The secret police and „provocations” in 1957 Spring] *Betekintő* 1, no. 2 (2007). Retrieved from: http://www.betekinto.hu/sites/default/files/betekinto-szamok/2007_2_krahulcsan.pdf 28 April 2021.

^43^
Only 8 secondary grammar schools remained in the maintenance of the Roman Catholic Church after the nationalisation, one in Kecskemét. R. Braham, *Education in the Hungarian People’s Republic* (Washington: US Government Printing Office, 1970), p. 77. Retrieved from: https://files.eric.ed.gov/fulltext/ED039635.pdf 28 April 2021.

^44^
A kecskeméti honvéd tiszti karhatalom története [The history of Kecskemét armed forces] HL MN KGY F. 1956, 11. d. 3. ő. e.; R. Kerepeszki, ʻ”Az egyetem nem a tudomány, hanem a reakció és a jobboldali gondolat fellegvára”. Jobboldalinak minősített egyetemi szervezkedések 1945–1956 között’ [„University is not the citadel of sciences, but reaction and right wing ideology”. Uiversity organizations, qualified as right wing orientated between 1945 and 1956] in *Búvópatakok. A jobboldal és az állambiztonság 1945–1989* [Underground streams: The right wing ad secret police, 1945-1989]. ed. K. Ungváry (Budapest: 1956-os Intézet-Jaffa, 2013), 293-319. There was a university based underground group in Szeged that time, called Turul Guard (Turul Gárda – turul bird was a symbol of the ancient Hungarians and the national identity), further investigations needed to see suspected connections between Szeged and Kecskemét.

^45^
P. Kenez, *The birth of the propaganda state. Soviet methods of mass mobilization, 1917–1929* (Cambridge: Cambridge University Press, 1985); M. Rolf, *Soviet Mass Festivals, 1917–1991* (Pittsburgh: University of Pittsburgh Press, 2013). The Hungarian communist practice of mobilizing and manipulating masses followed the Soviet role model, methods and tools are very similar in the hands of Party members or revolutionists.

^46^
A Szegedi Honvéd tiszti karhatalom története [The history of armed forces in Szeged] HL MN KGY F. 1956, 11. d. 10. ő. e.

^47^
A miskolci „Tizes honvéd” … Language instructions and religious education were the two main battlefields in schools, Mindszenty, the head of Catholic Church was an emblematic person of anti-communism.

^48^
I. Honvéd Forradalmi Ezred Parancsnokság története [The history of the Commandant of the 1 ^st^ Revolutionary Regiment] HL MN KGY F. 1956, 10. d. 1. ő. e. Through the Regiment’s naming, the new power tried to sequester and confiscate the meaning and connotations of revolution from the real rebels – the battle continued on a verbal level.

^49^
Ibid.

^50^
J. Kornai, ʻ1956 in Hungary: as I saw it then and as I see it now’ *Public Choice* 187, no 1-2 (2020): 15-26. In his essay, the famous Hungarian economist tried to exceed the traditional dichotomy of revolution vs. counter-revolution, including the ideas and bias related to it.

^51^
I. Honvéd Forradalmi Ezred Parancsnokság története [The history of the Commandant of the 1 ^st^ Revolutionary Regiment] HL MN KGY F. 1956, 10. d. 1. ő. e.; 2. Forradalmi Ezred története [The history of 2 ^nd^ Revolutionary Regiment] HL MN KGY F. 1956, 10. d. 2. ő.e.; Budai III. Forradalmi Karhatalmi ezred I. ker. századának története [The History of the Company in 1 ^st^ District, Buda, 3rd Regiment] HL MN KGY F. 1956, 10. d. 5. ő. e.

^52^
2. Forradalmi Ezred története [The history of 2 ^nd^ Revolutionary Regiment] HL MN KGY F. 1956, 10. d. 2. ő.e.

^53^
Ibid.

^54^
I. Honvéd Forradalmi Ezred … 65. [1 ^st^ Hungarian Revolutionary Regiment]

^55^
Budai III. Forradalmi Karhatalmi ezred I. ker. századának története [The History of the Company in 1 ^st^ District, Buda, 3rd Regiment] HL MN KGY F. 1956, 10. d. 5. ő. e.

^56^
P. T. Webb, ʻRe-mapping power in educational micropolitics’ *Critical Studies in Education* 49, no. 2 (2008): 127-142.

^57^
P. Borenich, ʻTörténetem … a Veszprém Megyei Rendőr-főkapitányság Politikai Nyomozó Osztályával (1957) [My story … With the Department of the Secret Police at the Veszprém County Police Headquarters (1957)]. Manuscript. Retrieved from: https://borenichpeter.hu/dokumentum-osszeallitasok-tenyfeltaro-riportok/83-tortenetem-a-veszprem-megyei-rendor-fokapitanysag-politikai-nyomozo-osztalyaval 28 April 2021. Security police, armed forces, police forces or state security have been mixing concepts in the discourses since then, because of overlapping functions and structures, especially in the years of 1956 and 1957.

^58^
Ibid.

^59^
Budai III. Forradalmi Karhatalmi ezred … I. Vida ed., *Diák- és ifjúsági mozgalmak, 1956–1958* [Students Movements, 1956–1958] (Budapest: Gondolat, 2007), 35., 78-79. The location is maybe the Central Dormitory of the BME on the András Hess square, an iconic location related to the student movement.

^60^
Budai III. Forradalmi Karhatalmi ezred …

^61^
Ibid.

^62^
2. Forradalmi Ezred századainak története [The history of Battalions of the 2 ^nd^ Revolutionary Regiment] HL MN KGY F. 1956, 10. d. 3. ő. e.

^63^
Ibid.

