# Supplementary material for: Violence in the Education: The Post-Revolutionary Situation in Hungary (1956-1957)
Source: F1000Res. 2023 Sep 28;12:1244. [Version 1] doi: 10.12688/f1000research.140928.1 (PMC10719558; doi:10.12688/f1000research.140928.1)
Supplement: Peer review history

# Reviewer report: Reviewer response for version 1

**DOI:** 10.5256/f1000research.154336.r211271

**Published:** 2023-12-13

**Authors:** Iveta Kestere

**Affiliations:** 1 Faculty of Education, Psychology and Art, University of Latvia, Riga, Latvia

The article ‘Violence in the Education: The Post-Revolutionary Situation in Hungary (1956-1957)’ analyses an under-researched topic, namely the repressions in the field of education after the failure of the Hungarian anti-Soviet revolution of 1956. Lajos Somogyvári's source material is professionally retrieved, it is unique, it reveals to the reader the mechanism of how the totalitarian power dealt with its opponents by humiliating and physically influencing them. This study is another important contribution to the history of 20th century’s totalitarianisms. The focus of the study is revealed by the four research questions, the methodology and the sources used are clearly presented. The author's ethical position, with respect for the actors of recent history, is to be commended.

 I have just some suggestions to make the text better understandable for an international audience. First and foremost, I would recommend that the author prefaces the article with more background information on the events in Hungary in 1956-57, such as how Hungary came to the Soviet sphere of influence, why the revolution or uprising of 1956 against the existing government started, what forces initiated and led the uprising, what the rebels wanted, and how the uprising was put down.

 Secondly, I suggest to consider some concepts that the reader may not be familiar with: for example, “After the end of socialism” (p.3) Here I suggest using a more familiar term, such as the collapse of the Soviet Union or the collapse of the Socialist Blok. “Reformer communists” (p.5), “Party apparatchiks” (p.6) and “pioneer patrols” (p.10): the text should explain what they were. “…the loyal members of the new power” (p.8) What is meant by "the new power" here?

 Third, I think that the rich material gives rise to broader conclusions that should be more clearly linked to the research questions on page 4. The valuable thoughts expressed in the conclusion section of the summary may also be useful.

 Admittedly, the meaning of some expressions in the text is not very clear: “came from the contemporary authentic party and military documents” (p.3), “the last stage of socialization has been depicted in agriculture” (p.3), “the special armed troops visiting schools armed groups”(p.3.), “agents of real communism” (p.5), “real hard-core communism” (p.5), “between form teachers and students” (p.9).

 As there are some other parts of the text that are difficult to read or can be misunderstood, perhaps the services of a proofreading could be helpful here.

 Some technical notes. I recommend that links to archive material should not be included in the text, but only in footnotes. Is Figure 1 (p.5) necessary? The research process is well described in the text and does not appear to be so complex as to need a scheme.

 With a few shortcomings addressed, the article by Lajos Somogyvári will certainly be a valuable asset for the international audience of researchers in recent history and totalitarianism, as well as historians of education.

Is the case presented with sufficient detail to be useful for teaching or other practitioners?

Yes

Is the work clearly and accurately presented and does it cite the current literature?

Yes

If applicable, is the statistical analysis and its interpretation appropriate?

Not applicable

Are all the source data underlying the results available to ensure full reproducibility?

Yes

Are the conclusions drawn adequately supported by the results?

Partly

Is the background of the case’s history and progression described in sufficient detail?

Partly

Reviewer Expertise:

History of education, particularly, history of education in the Soviet Union, education of New Soviet Man, visual propaganda in education, as well as childhood representations within museums.

I confirm that I have read this submission and believe that I have an appropriate level of expertise to confirm that it is of an acceptable scientific standard, however I have significant reservations, as outlined above.

# Reviewer report: Reviewer response for version 1

**DOI:** 10.5256/f1000research.154336.r211272

**Published:** 2023-12-13

**Authors:** Michal Šimáně

**Affiliations:** 1 Department of Social Sciences, Mendel University, Brno, Czech Republic

The author's text reveals little or completely unknown facts from a critical period of contemporary Hungarian history. Through the historiographical approach of the history of everyday life, he provides some insights into the shape of education not only in the short period of the Hungarian Revolution, but especially in the period that followed the suppression of this revolution. Specifically, it traces the approximate period from the autumn of 1956 to the spring and summer of 1957. The topic may seem marginal from this point of view, but from the point of view of Hungarian history of education, it is one of the neuralgic points and therefore represents a very specific and significant topic. This is evidenced by the words of the author, who states in his text that even after more than half of a century since the events described, it is still a very sensitive issue for many (and not only for the witnesses of these events). Until now, it has essentially been a kind of a taboo. For this reason, I see the publication of the results of research on this topic as very beneficial. On the basis of the information presented in the text, I even believe that the study may contribute to a certain correction of the view of the issue that has been presented so far in Hungarian history of education. For this reason, the contribution of the text can be seen as very crucial as well. The paper is also valuable with regard to the sources and methodology used. In fact, the author works with sources that he discovered by chance and about which nothing has been known before. After all, without this "luck" this study could not have been produced.

 I also appreciate the fact that the author's methodological description is very extensive. This gives the reader a detailed insight into the methodological "kitchen" of the author and at the same time they can follow step by step how the author worked with different types of sources and how the whole text was created. The very detailed methodological description may have been deliberate, because it also allows an implicit understanding of certain limitations found in the study; for example, a certain "fragmentation" of the themes that construct the main part of the text. The author does not, as the reader might expect, focus on one area of education (e.g. primary education) and deal with it in depth, but, given the currently available sources (of which, I assume, there were only a limited number), he gives an insight into the different levels of education and rather "shows" the different approaches and procedures the repressive regime of the time used for its purposes. This also causes a certain "episodicity" of some of the stories described, or it may constantly raise new questions for the reader, which the text, even considering the availability of sources, cannot answer. Therefore, the study can also be considered a first step towards further in-depth research on the topic. The question is whether it will be possible to find new sources or living witnesses who would be willing to share with us more about the topic. This fact, among other things, raises another question. Namely, whether the author tried to find such witnesses himself and if so, how successful he was in this matter. From the text, unless I have missed something, this is not entirely clear.

 The research results themselves are presented in the text in a very appropriate way. They are very suggestive and give the reader a very accurate picture of the everyday reality of the education sector in the period under review and the approaches and procedures that the repressive authorities chose to deal with the situations that arose. In this context, it is a bit of a pity that the author does not also attempt a more general view and summary in the conclusion. In fact, the results presented in the text not only illustrate the situation prevailing in the education system in Hungary at the turn of 1956 and 1957, but de facto show the elements typical of the functioning of totalitarian regimes. In other words, in this light, the text also extends beyond the borders of Hungary, thus undoubtedly acquiring an international dimension in addition to a regional one.

Is the case presented with sufficient detail to be useful for teaching or other practitioners?

Yes

Is the work clearly and accurately presented and does it cite the current literature?

Yes

If applicable, is the statistical analysis and its interpretation appropriate?

Not applicable

Are all the source data underlying the results available to ensure full reproducibility?

Yes

Are the conclusions drawn adequately supported by the results?

Yes

Is the background of the case’s history and progression described in sufficient detail?

Yes

Reviewer Expertise:

History of Education, Pedagogy, History of Everyday Life

I confirm that I have read this submission and believe that I have an appropriate level of expertise to confirm that it is of an acceptable scientific standard.

# Reviewer report: Reviewer response for version 1

**DOI:** 10.5256/f1000research.154336.r211274

**Published:** 2023-11-17

**Authors:** Antonio Francisco Canales Serrano

**Affiliations:** 1 Universidad Complutense, Madrid, Spain

The article deals with a very interesting topic and makes a relevant contribution to the field of study of the history of education and especially to the history of education under dictatorships. 

 The use of previously unexplored sources, as well as their variety, is remarkable.

 I would like to make a single suggestion to the author. Is "violence" really the best term for the title? Wouldn't "repression and control" be preferable? Violence refers primarily to the use of physical force and this is not strictly the case here.

Is the case presented with sufficient detail to be useful for teaching or other practitioners?

Yes

Is the work clearly and accurately presented and does it cite the current literature?

Yes

If applicable, is the statistical analysis and its interpretation appropriate?

Not applicable

Are all the source data underlying the results available to ensure full reproducibility?

Yes

Are the conclusions drawn adequately supported by the results?

Yes

Is the background of the case’s history and progression described in sufficient detail?

Yes

Reviewer Expertise:

History of Education

I confirm that I have read this submission and believe that I have an appropriate level of expertise to confirm that it is of an acceptable scientific standard.

# Response

**Published:** 2023-11-17

**Authors:** Lajos Somogyvari

**Affiliations:** Education Sciences, University of Pannonia, Veszprém, Hungary, Hungary

Yes, this is a good point: violence is a too narrow term in this sense, so I will change the title to: Repression and Control in the Education...,

 Just wait for the next reviews and collect their suggestions.

 Thanks!

 Lajos
